# The Indirect Path From Mindful Parenting to Emotional Problems in Adolescents: The Role of Maternal Warmth and Adolescents’ Mindfulness

**DOI:** 10.3389/fpsyg.2018.00546

**Published:** 2018-04-13

**Authors:** Yuyin Wang, Yiying Liang, Linlin Fan, Kexiu Lin, Xiaolin Xie, Junhao Pan, Hui Zhou

**Affiliations:** Department of Psychology, Sun Yat-sen University, Guangzhou, China

**Keywords:** mindful parenting, warmth, mindfulness, emotional problems, indirect effect

## Abstract

Mindfulness has been demonstrated to have positive effects on children’s emotional functioning, and adaptive parenting practices are associated with fewer emotional problems. However, the association between mindful parenting and adolescent emotional problems has not been studied much. In the current study, the indirect path from mindful parenting to adolescent emotional problems was examined, with maternal warmth and adolescent dispositional mindfulness as potential mediators. A sample of 168 mother–child dyads participated in this study. A serial indirect effects model showed mother’s mindful parenting could decrease adolescent emotional problems through adolescent’s perceived maternal warmth and their dispositional mindfulness. Findings of this study imply that intervention in mindful parenting may have benefits for adolescents’ emotional problems through enhancing maternal warmth and children’s trait mindfulness.

## Introduction

Mindfulness has been demonstrated to be beneficial in various kinds of psychopathology, such as anxiety and depression (Hofmann et al., 2010), pain (Song et al., 2014; Bawa et al., 2015), substance abuse (Tang et al., 2015), and various physical illnesses (Aucoin et al., 2014). Over the past decade, integrating mindfulness into parenting has received increasing attention. Researchers have aimed to explore the effect of mindful parenting on different developmental outcomes (Tak et al., 2015; Medeiros et al., 2016). Adolescence is a transitional period in life, accompanied by cognitive, physiological, and emotional changes (e.g., Cameron, 2004). Together with these changes, emotional problems are highly prevalent among adolescents, which significantly interfere with their daily functioning (e.g., Van Oort et al., 2009; Salk et al., 2016). Previous studies have examined the association between traditional parenting practices (such as parental control, rejection, and warmth) and emotional problems (for a meta-analysis concerning depression, see McLeod et al., 2007a; regarding anxiety, see McLeod et al., 2007b), but little is known about the role of mindful parenting. Therefore, the aim of the current study is to gain more insight into the association between mindful parenting and adolescents’ emotional problems.

Mindful parenting was first proposed by Kabat-Zinn and Kabat-Zinn (1997) and was broadly defined as fostering everyday mindfulness in the context of parent–child interaction (Duncan et al., 2009). Although mindfulness practice mainly focuses on intrapersonal processes, which helps individuals change their internal state, it can be applied to interpersonal processes as well (Coatsworth et al., 2010). In the interpersonal process of parenting, Duncan et al. (2009) have proposed that mindful parenting comprises five key dimensions: listening with full attention; non-judgmental acceptance of self and child; emotional awareness of self and child; self-regulation in the parenting relationship; and compassion for self and the child.

The adoption of this non-judgmental, non-overreactive, and compassionate approach to the interpersonal process of parenting is hypothesized to bring positive outcomes for youth. Several studies have attempted to examine the hypothesis and revealed that mindful parenting was negatively associated with adolescents’ symptoms, although the correlations were quite small, especially for those studies using multi-informants (Geurtzen et al., 2015; Tak et al., 2015; Turpyn and Chaplin, 2016). Geurtzen et al. (2015) found that parent-reported mindful parenting only weakly correlated with adolescent-reported anxiety and depression symptoms. In another short-term longitudinal study, parent-reported mindful parenting dimensions could not predict adolescent-reported depression symptom 6 months later, although mindful parenting was negatively correlated with adolescents’ depression at the first survey (Tak et al., 2015). Therefore, to date the evidence supporting a direct association between mindful parenting and adolescent emotional problems is quite limited. It is plausible to hypothesize that mindful parenting might indirectly influence children’s outcomes through other variables.

Duncan et al. (2009) has proposed a theoretical model which try to explain the potential mechanism of the effect of mindful parenting on youth outcomes. Their model elucidated that mindful parenting as a meta-parenting construct could promote other parenting practices and parent–child affection and finally reduce problematic youth outcomes and increase positive youth outcomes. Several studies partly support this theoretical model. For example, Parent et al. (2016b) found mindful parenting could influence parenting practices and then have an impact on internalizing and externalizing problems among youth. Turpyn and Chaplin (2016) also revealed that mindful parenting could influence adolescent substance use through shared positive emotion. However, more studies are needed to investigate the potential pathways from mindful parenting to children’s outcomes.

Traditional parenting practices have been theoretically proposed to play a bridging role between mindful parenting and children’s outcomes (Duncan et al., 2009). When parents integrate mindfulness into the parenting process, they may have more awareness about their parenting practices. Correlational studies do find a significant association between mindful parenting and traditional parenting practices (Geurtzen et al., 2015; Parent et al., 2016a). Furthermore, the indirect effect of mindful parenting on children’s outcome through traditional parenting practices has been found across different developmental stages (Parent et al., 2016b). However, in these studies, mindful parenting and traditional parenting practices were both reported by parents. Whether this positive effect on parenting practice could be perceived by children remains poorly investigated. Children’s perceived parenting practice is more important in predicting their behavior and promoting social development (e.g., Barber, 1996; Gonzales et al., 1996). Parents who have high levels of mindful parenting could listen to their children with full attention and have compassion for the children as well as themselves. Therefore, it could be hypothesized that children would perceive more positive parenting practices, such as parental warmth, when parents have high levels of mindful parenting. As a primary dimension of parenting, the importance of parental warmth in reducing children’s internalizing problems, such as anxiety and depression, has been repeatedly supported (e.g., McLeod et al., 2007a,b). Thus, perceived parental warmth might play an important role between mindful parenting and children’s emotional problems.

Another possible factor that’s not included in Duncan et al.’s (2009) theoretical model in explaining the mechanism of mindful parenting is children’s resilience traits, such as children’s dispositional mindfulness. Dispositional mindfulness refers to the trait level of an individual’s mindful ability, which could be defined as a non-judgmental, present-centered awareness (Bishop et al., 2004). Increasing studies suggest mindfulness-based interventions are beneficial for youth, including children’s emotional problems (for a review, see Felver et al., 2016). However, although multiple intervention strategies are applied for increasing dispositional mindfulness, little is known about the development of mindfulness, or the process by which parents could cultivate this trait in their offspring (Roeser and Eccles, 2015). When parents take a mindful approach in the context of parenting, children may learn to be mindful directly through the social learning processes or indirectly through other positive parenting practices that are influenced by mindful parenting. Therefore, it could be hypothesized that children’s dispositional mindfulness could be one of the mechanism factors of mindful parenting.

**FIGURE 1:**
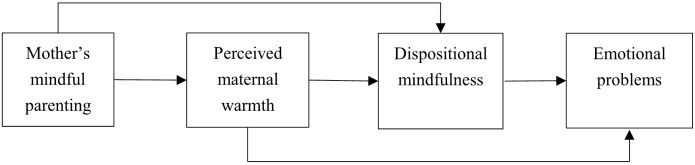
Theoretical model for delineating the indirect influence of mother’s mindful parenting on children’s emotional problems.

Given the paucity of studies explaining how mindful parenting is linked to better adjusted children’s outcomes, the current study aims to bridge this gap by bringing in positive parenting practice (warmth) and children’s dispositional mindfulness in explaining the mechanism linking mindful parenting to children’s emotional problems. An indirect pathway from maternal mindful parenting to adolescents’ emotional problems was hypothesized, with children’s perceived maternal warmth and dispositional mindfulness as serial mediators (**Figure 1**). Moreover, since most studies about mindful parenting were conducted in Western cultures, little is known about its effects in Chinese families. Previous studies have suggested that parenting is influenced by the social norms and values in a culture (Goodnow, 1985). Similar parenting practices may have different effects on children in different cultures (Leung et al., 1998). Thus, it is of great importance to explore mindful parenting beyond Western cultures.

In addition, mother–child dyads were used in the current study. Previous research that investigated the relationship between mindful parenting and parenting practice has mainly focused on parent-reported data (e.g., Geurtzen et al., 2015; Parent et al., 2016a), which may increase the risk of common method bias. In the current study, parenting practice was reported by the child, which was specified as children’s perceived maternal warmth, as well as children’s own dispositional mindfulness. Mindful parenting was reported by the mother.

## Materials and Methods

### Participants

One hundred and sixty-eight mother–child dyads were recruited in this study. The children sample consisted of slightly more girls (56%) than boys (44%), with the age ranging from 11 to 14 years (*M* = 12.89, *SD* = 0.56). The mothers were between the ages of 36 and 50 years (*M* = 42.45, *SD* = 3.14). With regard to educational level, 28 (26.7%) received middle school or below education, 112 (68.7%) had completed undergraduate studies, 23 (14.1%) had completed postgraduate studies, and 5 did not report their education level.

### Measures

#### Mindful Parenting

Mindful parenting was assessed by the Interpersonal Mindfulness in Parenting scale (IM-P; Duncan, 2007). This scale consists of 10 items reflecting parents’ ability to maintain (1) awareness and present-centered attention during parent–child interactions (e.g., “I notice how changes in my child’s mood affect my mood.”), (2) non-judgmental acceptance of their children’s thoughts and emotion (e.g., “I listen carefully to my child’s ideas, even when I disagree with them.”), and (3) the ability to regulate their reactivity to their children (e.g., “When I’m upset with my child, I notice how I am feeling before I take action.”). The Chinese version of the IM-P was translated from the original English version by three experts, back-translated by an English native speaker who is fluent in Chinese, and then adjusted for cultural adaptation. Mothers were asked to respond to the items on a five-point Likert scale. The mean was calculated, with higher scores reflecting higher levels of mindful parenting. The Cronbach’s alpha coefficient in this study was 0.660.

#### Children’s Perceived Maternal Warmth

A subscale of the Parental Acceptance-Rejection Questionnaire (PARQ) – Short Form was used to assess children’s perceived maternal warmth (Rohner and Khaleque, 2005). This scale has been used in several countries, including China (Putnick et al., 2012). The PARQ contains 24 items and four scales, including perceived warmth/affection, perceived hostility/aggression, perceived indifference/neglect, and perceived undifferentiated rejection. In this study, the perceived warmth/affection subscale was used (e.g., “My mother makes me feel wanted and needed.”). This subscale consists of 7 items. Children were asked to rate the items on a seven-point Likert scale according to their experience with their mothers. The mean was calculated, with higher scores indicating higher levels of perceived warmth and affection. The Cronbach’s alpha coefficient in this study was 0.930.

#### Mindfulness Traits of Children

Children’s mindfulness was assessed by the Chinese version of the Mindful Attention Awareness Scale (MASS) (Black et al., 2012; Deng et al., 2012). The scale contains 15 items (e.g., “I find it difficult to stay focused on what’s happening in the present.”). Children were asked to rate their agreement with these items on a six-point Likert scale. The mean was calculated, with higher scores representing higher levels of trait mindfulness. The Cronbach’s alpha coefficient in this study was 0.833.

#### Emotional Problems

Emotional problems were assessed by the emotional problems subscale of the Chinese version of the Strengths and Difficulties Questionnaire (SDQ) (Goodman, 1997; Kou et al., 2007). The emotional problems subscale contains five items (e.g., “I am often unhappy, downhearted or tearful.”). Children were asked to rate each item on a three-point Likert scale according to their situation. The mean was calculated, with higher scores representing higher levels of emotional problems. In this study, the Cronbach’s alpha coefficient was 0.760.

### Procedure

The sample was recruited from a public middle school in south China. Students’ mothers were contacted first through the school-parent online communication system. A letter explaining the study and the electronic edition of an informed consent form were sent to mothers through the system at the same time. After online consent was obtained from the mothers, the students were asked to complete the questionnaires in class. The mother-reported questionnaires were taken by the students to their mothers to complete at home and return to teachers or research assistants one or 2 days later. In total, 402 pairs of questionnaires were distributed, of which 298 (74.1%) student questionnaires and 234 (58.2%) mother questionnaires were returned. Among them, 168 dyads could be paired according to students’ names. Therefore, the final sample was composed of 168 mother–child dyads.

### Data Analytic Plan

All statistical analyses were conducted using SPSS 21.0 and Mplus 7.0 (Muthén and Muthén, 1998–2010). Missing data were treated as missing at random and Full Information Maximum Likelihood (FIML; Schafer and Graham, 2002) was used in Mplus. Independent-samples *t*-tests, bivariate correlations, and one-way ANOVA were conducted to investigate the effects of demographic variables (children’s gender and age, mothers’ educational level) on the primary outcome variables. Those demographic variables that had significant effects on outcome variables would be controlled in the following path analysis (Atinc et al., 2012).

A serial indirect effects model was conducted to test the hypothesized model shown in **Figure 1**, which emphasizes testing indirect effects (Hayes, 2013). Mplus version 7 was used to evaluate individual pathways and indirect effects. Ten thousand bootstrap samples were used in each test of the indirect effect. Bias-corrected bootstrap confidence intervals for indirect effects were contained in each model. The indirect effect was declared as significant if the corresponding 95% bias-corrected bootstrap standardized confidence interval did not contain zero.

## Results

### Preliminary Analysis

Normality of the data was examined first. The skewness values were from -0.697 to 0.026 and the kurtosis values were from -0.749 to 0.806; their absolute values were less than 2 and 7, respectively. Thus, the data could be considered as normally distributed (Finney and DiStefano, 2006).

Before testing the hypothesized path model, demographic variables were analyzed to identify the potential covariates that should be controlled in the path model. Correlation analysis showed children’s age was not correlated with any key study variables (*r* = 0.021 for mindful parenting, *r* = 0.049 for maternal warmth, *r* = -0.124 for dispositional mindfulness, *r* = 0.056 for emotional problems, all *p* > 0.05). No significant difference was found between boys’ and girls’ mindful parenting [*t*(164) = 1.298, *p* > 0.05], maternal warmth [*t*(164) = -0.903, *p* > 0.05], dispositional mindfulness [*t*(164) = 0.418, *p* > 0.05], and emotional problems [*t*(166) = -0.943, *p* > 0.05]. None of the study variables significantly differed by mothers’ educational level, [*F*(3,160) = 0.844 for mindful parenting, *F*(3,160) = 0.425 for maternal warmth, *F*(3,160) = 0.686 for dispositional mindfulness, and *F*(3,162) = 0.311 for emotional problems, all *p*s > 0.05]. Therefore, no demographic variables were included as covariates in the path model. The correlations among all key study variables are shown in **Table 1**.

**Table 1:** Descriptive statistics and correlations.

	*M* (*SD*)	Mindful parenting	Perceived maternal warmth	Dispositional mindfulness	Emotional problems
(1) Mindful parenting	3.84 (0.53)	1			
(2) Perceived maternal warmth	5.12 (1.48)	0.194^∗^	1		
(3) Dispositional mindfulness	4.16 (0.83)	0.027	0.331^∗∗^	1	
(4) Emotion problem	1.53 (0.48)	-0.025	-0.158^∗^	-0.364^∗∗^	1


*^∗^p < 0.05, ^∗∗^p < 0.01.*

### Serial Indirect Effects Model Analysis

The hypothesized model was examined first [χ^2^(1) = 0.016 (*p* = 0.899), CFI = 1.000, TLI = 1.139, RMSEA < 0.0001 (90%CI (0.000, 0.092), *p* = 0.918), SRMR = 0.002]. Several hypothesized individual pathways were not significant, including mindful parenting to dispositional mindfulness (β = -0.043, *p* = 0.592) and perceived maternal warmth to emotional problems (β = -0.046, *p* = 0.579). The other significant pathways are shown in **Table 2**. To simplify the model, all insignificant individual pathways were excluded in the final model.

**Table 2:** Serial mediation models.

Mindful parenting → Perceived maternal warmth → Dispositional mindfulness → Emotional problems									
	M1			M2			Y		
	Perceived maternal warmth			Dispositional			Emotional problems		
	β	*SE*	*p*	β	*SE*	*p*	β	*SE*	*p*
X (mindful parenting)	0.19	0.08	<0.05	–	–	–	–	–	–
M1 (perceived maternal warmth)	–	–	–	0.33	0.08	<0.01	–	–	–
M2 (dispositional mindfulness)	–	–	–	–	–	–	-0.36	0.10	<0.01
Indirect effects									
*Estimate* = -0.02, *SE* = 0.01, 95% CI (-0.05, -0.01)									

**FIGURE 2:**
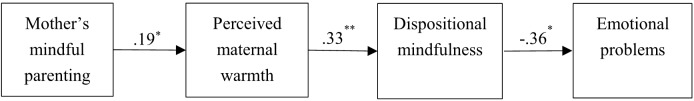
Final path model for analyzing indirect influence of mother’s mindful parenting on children’s emotional problems. The numbers in the figure represent the standardized coefficients. Model fit was good: χ^2^(3) = 0.709 (*p* = 0.871), CFI = 1.000, TLI = 1.108, RMSEA < 0.0001 (90%CI (0.000, 0.066), *p* = 0.925), SRMR = 0.016, AIC = 1188.855, BIC = 1216.863. The presented model is the model with all non-significant individual pathways excluded. ^∗^*p* < 0.05, ^∗∗^*p* < 0.01.

**Figure 2** depicts the final model. As expected, mindful parenting had significant positive effects on children’s perceived maternal warmth. However, mindful parenting did not have any direct effect on dispositional mindfulness. The pathway from perceived maternal warmth to emotional problems was not significant; thus, mindful parenting could not affect children’s emotional problems through perceived maternal warmth. However, there were significant indirect effects in mother-reported mindful parenting and children’s self-reported emotional problems through perceived maternal warmth and dispositional mindfulness (*Estimate* = -0.02, *SE* = 0.01, 95% CI [-0.05, -0.01]). The total *R* square for emotional problems was 13.1%.

## Discussion

This study aimed to explore the association between mindful parenting and emotional problems in adolescents, and the potential indirect effect pathway. The roles of maternal warmth and dispositional mindfulness were examined.

Consistent with our hypothesis, maternal mindful parenting was not directly correlated with adolescents’ emotional problems. In other studies using multi-informants, some dimensions of mindful parenting were found to be weakly but significantly correlated with emotional problems (Geurtzen et al., 2015; Tak et al., 2015). The significant correlations may partly have been due to the large sample sizes they used (for example more than 1000 adolescent in Geurtzen et al.’s study). There have also been findings consistent with our results that parent-reported mindful parenting was not directly associated with children-reported well-being (Medeiros et al., 2016). The hypothesis of an indirect association between mindful parenting and emotional problems is supported in our sample.

Although a direct association between mindful parenting and emotional problems was absent, mindful parenting could still affect emotional problems. According to Duncan et al.’s (2009) theoretical model, mindful parenting could influence children’s developmental outcomes through parenting practices. Therefore, specific parenting practices have been considered as mediators (Parent et al., 2016b). However, in that study, parent-reported parenting practices were investigated, and little is known about whether the positive influence of mindful parenting could be perceived by children. The findings from this study provide preliminary support that children’s perception of maternal warmth is positively associated with levels of mothers’ mindful parenting. However, only maternal warmth was investigated in the present study. Further studies could explore other children’s perceived parenting practices.

Given the increasing evidence that supports the benefits of mindfulness, researchers have showed great interests in how to nurture mindfulness in children and youth (Greenberg and Harris, 2012). Previous studies mostly focused on the effect of mindfulness-based interventions on enhancing individuals’ dispositional mindfulness and further decreasing their emotional symptoms (for a meta-analysis, see Hofmann et al., 2010). This study preliminarily investigated the relationship between children’s dispositional mindfulness and family context variables. The findings revealed children’s dispositional mindfulness was not directly associated with mindful parenting, but could be nurtured in a mindful parenting family context through children’s perceived warmth.

A serial indirect effects model was used to examine the indirect pathway from mindful parenting to adolescents’ emotional problems with two sequential bridging variables. The results supported that mindful parenting could have beneficial effects on children’s emotional problems indirectly through perceived maternal warmth and children’s dispositional mindfulness. The indirect pathway partly supported Duncan et al.’s (2009) theoretical model and expanded it as well. Parenting practices were proposed as a mediator in their model, and maternal warmth as a specific parenting practice was examined. However, in their model, children’s positive traits, such as mindfulness, were treated as outcome variables. The findings from this study revealed that children’s dispositional mindfulness could act as a mediator as well, and totally mediated the correlation between maternal warmth and emotional problems. The results further implied that perhaps some children’s positive traits could be cultivated in a mindful family context and then bring about positive developmental outcomes. Further studies are needed to examine this point.

With the increasing attention on mindful parenting, exploring its effect on children’s development has great significance. The results from the current study and other studies repeatedly found mindful parenting has little direct association with adolescent problematic or positive outcomes (Tak et al., 2015; Medeiros et al., 2016). This supported the idea that mindful parenting should be regarded as a meta-parenting construct instead of a specific parenting practice (Duncan et al., 2009). Parents who adopt a mindful attitude in the parenting context are more aware about how they are parenting their children and are thus better able to choose appropriate methods and practices in interactions with their children. As shown in the current study, a warm, non-judgmental, and acceptant parenting context is more prone to facilitate and promote the development of dispositional mindfulness or other resilience traits in adolescents. Finally, consistent with other studies’ results, adolescents who are more mindful develop fewer psychosocial problems (e.g., Zelazo and Lyons, 2012; Felver et al., 2016). The current study is concerned with establishing a possible pathway that can show how mindfulness could benefit adolescent’s development.

There are several limitations in the present study that should be noted. First, the data is cross-sectional, so the serial indirect effects model tested in this study should be regarded as a preliminary investigation. Future research should utilize longitudinal designs to test the whole model so that the mechanism could be more robust. Second, only mothers were recruited in the current study, which reduced the generalizability of the findings to the whole family. Father samples are often underrepresented in child and adolescent research (Phares et al., 2005), but fathers must play an important role in socializing children, especially in adolescence (Parent et al., 2016b). In addition, the participants in the current study were recruited in an urban city. However, rural areas have different subcultures, such as child policies and SES (Yang, 1986; Mackerras, 2001). Third, only warmth was tested when investigating the traditional parenting practices. Mindful parenting as a meta-parenting construct was believed to influence many parenting practices. As Parent et al. (2016b) found, mindful parenting could influence positive parenting practices (expressions of warmth and affection, use of positive reinforcement, etc.) and negative parenting practices (reactive, coercive, etc.). However, the effect size of mindful parenting on positive parenting practices was much bigger than for negative parenting practices (Parent et al., 2016b). Therefore, the current study first examined one of the positive parenting practices. Further research could explore more specific parenting practices. Lastly, the measurement tool used in this study for assessing mindful parenting has not been validated yet in the Chinese population. We used the 10-item version, initially developed by Duncan (2007). Recently, the Interpersonal Mindfulness in Parenting questionnaire was validated in a Chinese sample in Hong Kong (Lo et al., in press). They revised the 31-item version of IM-P developed by Duncan et al. (2009). Their validated Chinese version of IM-P was not developed when we conducted the current study. We believe more robust results could have been generated with more reliable measures.

The current study also had several strengths and practical implications that should be noted. First, child–mother dyads were recruited in data reporting and each child was required to report the parenting practice s/he perceived. Therefore, it could be known how a mindfully parenting mother could influence her child’s perception of her parenting. Second, the findings of this study supported the importance of developing a mindful parenting intervention program (Bögels and Restifo, 2014), since mindful parenting could have direct and indirect effects on parenting and children’s outcomes. Third, dispositional mindfulness was preliminarily found to be associated with parenting variables. Mindfulness is repeatedly demonstrated to be a health-promoting trait (Brown and Ryan, 2003; Shapiro et al., 2006). Although it can be cultivated through mindfulness-based intervention, whether it can be cultivated through ordinary parenting is an important theoretical and practical question. This study sheds some light on this question to find that mindful parenting and maternal warmth could have positive effects on it.

## Ethics Statement

This study was carried out in accordance with the recommendations of the Institutional Review Board (IRB) of the Department of Psychology, Sun Yat-sen University, China, with written informed consent from all subjects in accordance with the Declaration of Helsinki. The protocol was approved by the Institutional Review Board (IRB) of the Department of Psychology, Sun Yat-sen University, China.

## Author Contributions

YW, JP, and HZ made important contributions in writing this paper. YW led the writing work and took the responsibility as first author. YL analyzed the data. LF, KL, and XX helped to collect the data.

## Conflict of Interest Statement

The authors declare that the research was conducted in the absence of any commercial or financial relationships that could be construed as a potential conflict of interest. The reviewer XL and handling Editor declared their shared affiliation.
